# Silencing LY6D Expression Inhibits Colon Cancer in Xenograft Mice and Regulates Colon Cancer Stem Cells’ Proliferation, Stemness, Invasion, and Apoptosis via the MAPK Pathway

**DOI:** 10.3390/molecules28237776

**Published:** 2023-11-25

**Authors:** Jinyue Duan, Yi Wang, Yuanyuan Chen, Yujue Wang, Qisen Li, Jinrui Liu, Changhao Fu, Chenyu Cao, Zhongyi Cong, Manman Su

**Affiliations:** 1 Department of Regenerative Medicine, School of Pharmaceutical Sciences, Jilin University, Changchun 130021, China; duanjy18@mails.jlu.edu.cn (J.D.); muyao1221@foxmail.com (Y.C.); yujue21@mails.jlu.edu.cn (Y.W.); 13805960948@163.com (Q.L.); jrliu22@mails.jlu.edu.cn (J.L.); caocy2820@163.com (C.C.);; 2 VA Palo Alto Health Care System, Medical School, Stanford University, Palo Alto, CA 94304, USA; denis-fu@hotmail.com

**Keywords:** colon cancer, cancer stem cells, MAPK, LY6D

## Abstract

This study explored the role of lymphocyte antigen 6 family member D (LY6D) in colon cancer stem cells’ (CCSCs) proliferation and invasion. LY6D was knocked down using siRNA, and the down-regulation of LY6D was verified using Western blotting. After LY6D knockdown, CCSCs’ proliferation, stemness, and invasion were suppressed, whereas apoptosis was increased. Gene Ontology (GO) enrichment analysis revealed that the differentially expressed genes (DEGs) between siLY6D and the negative control groups were significantly enriched in the cell–substrate adherens junction, focal adhesion, and cell–substrate junction terms. Meanwhile, the Kyoto Encyclopedia of Genes and Genomes (KEGG) enrichment analysis revealed that the DEGs were significantly enriched in the MAPK pathway. In addition, Western blotting results showed that pBRAF and pERK1/2, cascade kinases of the MAPK pathway, were significantly down-regulated after LY6D knockdown. In addition, nude mice xenograft experiments showed that the siLY6D treatment decreased tumor sizes and weights and improved tumor-bearing mice survival rates compared with the control group. In conclusion, these findings indicate that LY6D, which is highly expressed in CCSCs, is a key factor involved in tumor growth and development and might be a potential cancer marker and therapeutic target for colon cancer.

## 1. Introduction

Colon cancer (CC) is one of the most commonly diagnosed digestive system cancers worldwide. According to GLOBOCAN 2020, CC was the third, most diagnosed cancer and the second leading cause of global cancer-related deaths in 2020 [1]. Although there are several treatments for CC, such as surgery, radiation, and chemotherapy, alone or in combination, a standard therapeutic method for patients with CC is still undefined owing to the poor 5-year survival rate caused by recurrence and metastasis [2,3,4]. Although diet, microorganisms, and their metabolites are associated with CC, the detailed mechanisms of CC development remain unclear [5]. Therefore, elucidating the molecular mechanisms of CC is extremely important.

Cancer stem cells (CSCs) possess self-renewal, infinite cell proliferation, and metastasis capacity, which are the properties that cause cancer to be heterogenous and make cancer treatment challenging [6,7,8]. In recent years, CSCs have been shown to exist in a variety of solid tumors, including colon, breast, lung, liver, prostate tumors, and many other malignant cancers [9,10,11,12,13]. Colon cancer stem cells (CCSCs) are thought to drive CC initiation, proliferation, and metastasis [14,15]. Therefore, exploring the mechanisms through which CCSCs drive CC initiation is critical for determining a target for CC curative treatment.

The lymphocyte antigen-6 (LY6) family proteins were first discovered in mice [16]. Subsequently, human homologs of LY6 were isolated and found to be well conserved, with a wide range of expression patterns and critical functions [17]. In recent years, LY6 family proteins have been reported to be involved in the regulation of neuronal activity, cell proliferation, migration, cell–cell interaction, immune cell maturation, the progression of inflammation, and angiogenesis [18,19,20,21,22]. High expression of LY6 family members is reportedly associated with poor prognosis of some cancers. High expression of LY6E enhances the progression of CC [23,24]. High expression of LY6K promotes the progression of bladder, ovarian, and breast cancer [25,26,27,28,29,30]. LY6D has been identified as a tumor-associated antigen in prostate and basosquamous cancers [31,32]. 

LY6D (lymphocyte antigen 6 complex, locus D), also named E48, located on human chromosome 8 in the 8q24 region, is a GPI-anchored protein which is predicted on the cell surface and the plasma membrane [33]. At first, LY6D was identified as a specification marker of the earliest stage of lymphocyte B cells [33]. Subsequently, it was found that LY6D was expressed in transitional epithelial cells but not in columnar epithelial cells. So far, LY6D has been used as a marker of squamous cell differentiation [34,35]. In recent years, it has been found that LY6D has increased expression in some type of cancers and is related to cell adhesion [36,37]. It has been found that LY6D can be a prognostic factor for advanced prostate cancer, which is positively correlated with poor outcomes of patient survival [32,38]. However, the role of LY6D in CC is still unknown.

In our research, LY6D was found to have a higher expression in CCSCs than in CC cell lines; thus, whether LY6D contributes to the progression of CC, or regulates the proliferation, migration, and apoptosis of CCSCs, should be investigated. Our studies showed that the knockdown of LY6D can inhibit the proliferation, stemness, and invasion of CCSCs while promoting their apoptosis. Moreover, the MAPK cascade kinases, pBRAF and pERK1/2, were significantly down-regulated via LY6D knockdown. In addition, the nude mice xenograft experiments revealed that mice treated with siLY6D had a longer survival rate compared with the control group.

## 2. Results

### 2.1. LY6D Expression in CC and CCSCs’ Screening and Identification

**Figure 1:**
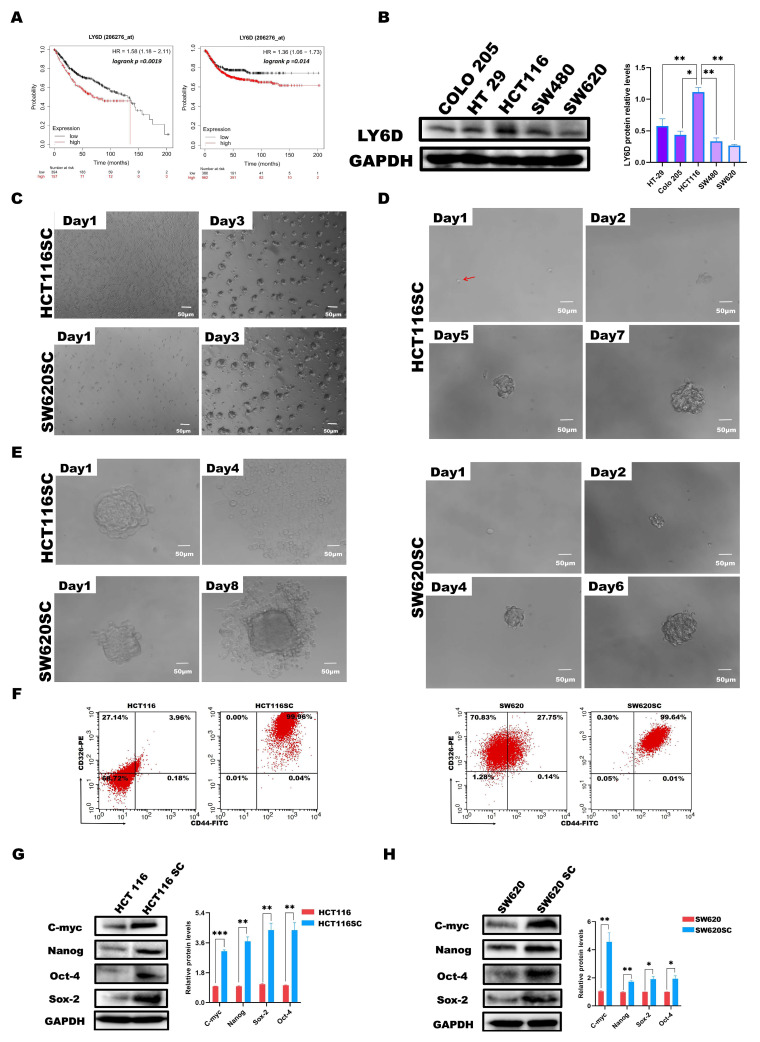
Effect of LY6D on the survival of patients with colon cancer (CC). Optical micrographs and identification of sorted colon cancer stem cells (CCSCs). (**A**) KM plotter survival curve showing the overall survival and the relapse-free survival of patients with CC based on high and low LY6D expression. (**B**) Western blot analysis of the relative protein levels of LY6D in various CC cell lines (* *p* < 0.05, ** *p* < 0.01, compared with HCT116 cells). (**C**) Culture of HCT116SCs and SW620SCs (sorted CCSCs from respective cell lines) in serum-free DMEM/F12 medium over three days. (**D**) Tumor sphere formation process from single cell of HCT116SCs’ and SW620SCs’ populations (The red arrow indicates the single-cell location). (**E**) Differentiation of CCSCs into adherent cells after induction with 10% FBS. (**F**) Flow cytometric analysis of the expression ratios of CD44 and CD326, comparing HCT116SCs and SW620SCs with HCT116 and SW620 cells. (**G**) The relative protein levels of c-Myc, Sox-2, Nanog, and Oct-4 in HCT116 and HCT116SCs’ populations were compared via Western blotting. (**H**) The relative protein levels of c-Myc, Sox-2, Nanog, and Oct-4 in SW620 and SW620SCs’ population compared via Western blotting; * *p* < 0.05, ** *p* < 0.01, *** *p* < 0.001.

By analyzing the Kaplan–Meier plotter database (https://kmplot.com/analysis/ (accessed on 19 May 2023)) [39,40], we found that LY6D was associated with poor prognosis in patients with CC; specifically, patients with higher LY6D expression had a worse overall survival and progression-free survival than patients with lower LY6D expression (Figure 1A). Subsequently, we explored the expression of LY6D in several different CC cell lines and found that it was expressed at the highest level in HCT116 cells and the lowest level in SW620 cells (Figure 1B). To detect the expression levels of LY6D in CCSCs, we sorted CCSCs from HCT116 and SW620 cell populations, which were referred to as HCT116SCs and SW620SCs, respectively. CD44 and CD326 have been identified previously as surface markers of CCSCs and are used to isolate the cells from CC cell populations [41,42,43,44]. In the present study, HCT116SCs and SW620SCs were isolated, and then the CCSCs were cultured in FBS-free DMEM/F12, of which the sorted CCSCs could form spheres after being cultured for three days (Figure 1C). In addition, even only one cell of the HCT116SC or SW620SC population was able to form a sphere (Figure 1D). Moreover, the spheres of HCT116SCs and SW620SCs retained their capacity to form adherent cells upon induction (Figure 1E). Moreover, the expression of CD44 and CD326 on the sorted CCSCs was evaluated via flow cytometry, and the proportions of CCSCs among the sorted cells were significantly higher than those among unsorted CC cells (Figure 1F). Western blotting analysis further revealed that the relative protein expression levels of c-Myc, Nanog, Sox-2, and Oct-4, which are recognized as putative stem cell markers, were significantly higher in CCSCs than in CC cells (Figure 1G,H).

### 2.2. LY6D Was Expressed at High Levels in CCSCs

**Figure 2:**
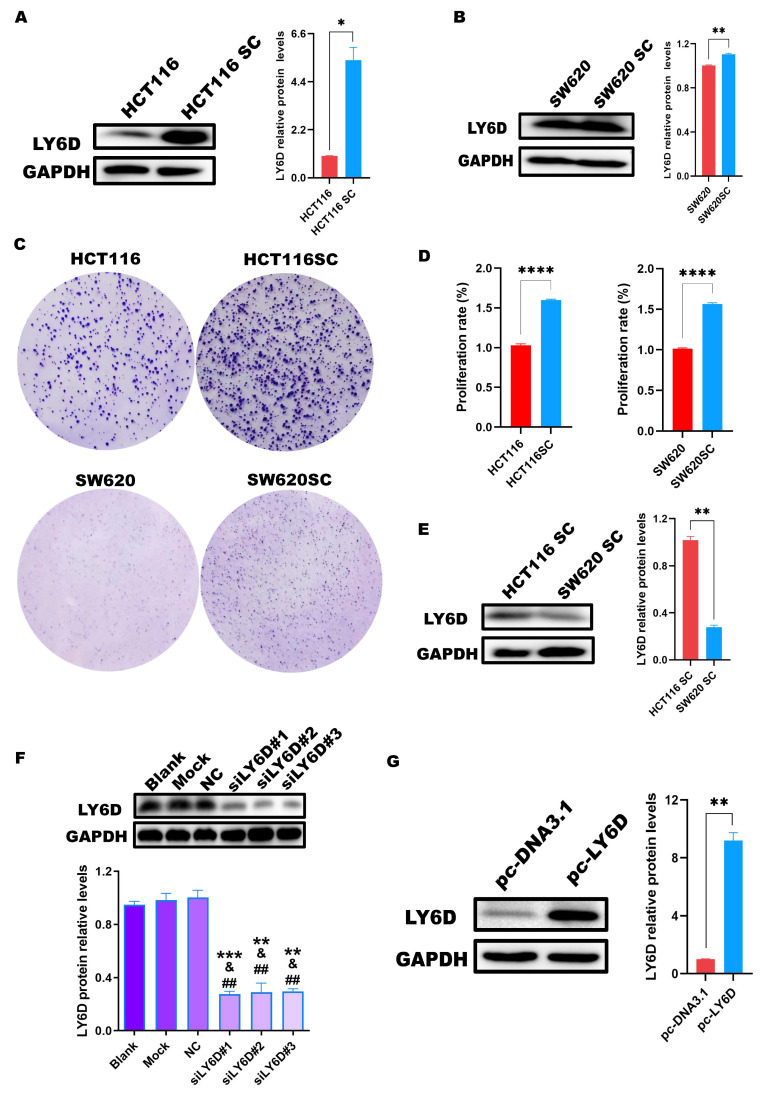
Expression of LY6D in colon cancer stem cells (CCSCs). (**A**) Western blotting results showing LY6D protein levels in HCT116 cells and HCT116SCs (stem cells) (* *p* < 0.05, compared with HCT116). (**B**) Western blotting results showing LY6D protein levels in SW620 cells and SW620SCs (stem cells) (** *p* < 0.01, compared with SW620). (**C**) Representative colony formation assay results capture of HCT116 vs. HCT116SC and SW620 vs. SW620SC. (**D**) MTS assay results showed the proliferation capability of HCT116 vs. HCT116SC and SW620 vs. SW620SC (**** *p* < 0.0001, compared with HCT116SC or SW620SC). (**E**) Western blotting results showing LY6D protein levels in HCT116SCs and SW620SCs (** *p* < 0.01, compared with HCT116SCs). (**F**) Western blotting results showing LY6D protein levels in HCT116SCs after siLY6D transfection for 48 h (** *p* < 0.01, *** *p* < 0.001, compared with blank; ^&^ *p* < 0.05, compared with mock; ^##^ *p* < 0.01, compared with negative control (NC)). (**G**) Western blotting results showing LY6D protein levels in SW620SCs after pc-DNA3.1-LY6D (pc-LY6D) transfection for 48 h (** *p* < 0.01, compared with pc-DNA3.1 transfection).

Next, we explored the expression of LY6D in HCT116 cells and HCT116SCs via Western blotting. According to the results, the expression in HCT116SCs was significantly higher than that in HCT116 cells (Figure 2A). We also compared the expression of LY6D in SW620 cells and SW620SCs, which showed that LY6D expression was significantly higher in SW620SCs than in SW620 cells (Figure 2B). In addition, the colony formation capacity and the proliferation capacity of HCT116SC and SW620SC were significantly higher than that of HCT116 and SW620, respectively (Figure 2C,D). Moreover, we compared the expression of LY6D in HCT116SCs and SW620SCs and observed that LY6D expression was significantly higher in HCT116SCs than in SW620SCs (Figure 2E). Therefore, to explore the effect of LY6D on the biological behavior of CCSCs, its expression in HCT116SCs was knocked down and its expression in SW620SCs was up-regulated. Western blotting results showed that after treatment with siLY6D#1, siLY6D#2, and siLY6D#3, LY6D protein expression was significantly knocked down compared with those in the blank, mock, and NC groups, respectively (Figure 2F). Western blotting results also showed that the LY6D protein could be overexpressed significantly after treatment with pc-LY6D when compared with the levels in the pc-DNA3.1 group (Figure 2G).

### 2.3. Down-Regulation of LY6D Expression Reduced HCT116SCs’ Proliferation, Invasion, and Stemness and Increased Apoptosis

**Figure 3:**
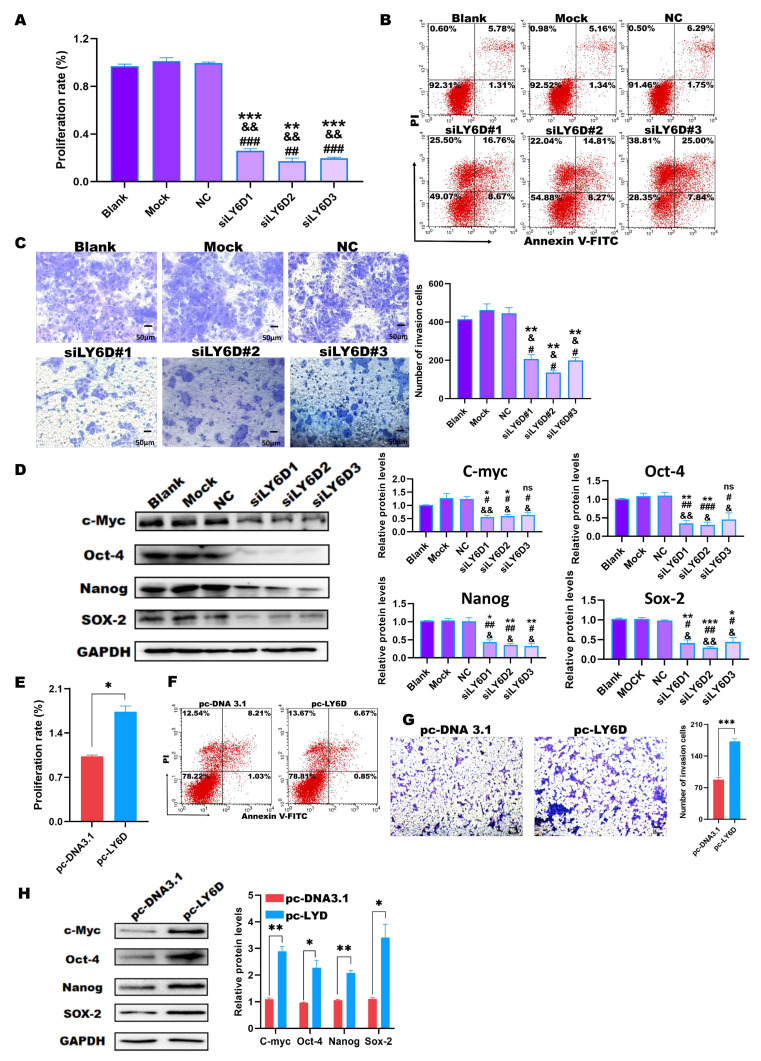
In vitro experiments to identify the relationship between LY6D and colon cancer stem cells (CSCCs) proliferation, apoptosis, and invasive capacity. (**A**) MTS assay showed the proliferation potential of HCT116SCs (stem cells) after siLY6D transfection for 48 h (** *p* < 0.01, *** *p* < 0.001, compared with blank; ^&&^ *p* < 0.01, compared with mock; ^##^ *p* < 0.01, ^###^ *p* < 0.001, compared with negative control (NC)). (**B**) Flow cytometric analysis of Annexin V/PI staining to determine the percentage of apoptotic HCT116SCs after siLY6D transfection for 48 h. (**C**) Representative photographs of HCT116SCs that passed through the Matrigel after siLY6D transfection for 48 h in the Transwell invasion assay (** *p* < 0.01, compared with blank; ^&^ *p* < 0.05, compared with mock; ^#^ *p* < 0.05, compared with NC). (**D**) Relative protein levels and normalization of c-Myc, Sox-2, Nanog, and Oct-4 detected via Western blotting after LY6D down-regulation (* *p* < 0.05, ** *p* < 0.01, *** *p* < 0.001, compared with blank; ^&^ *p* < 0.05, ^&&^ *p* < 0.01, compared with mock; ^#^ *p* < 0.05, ^##^ *p* < 0.01, ^###^ *p* < 0.001, compared with negative control (NC)). (**E**) MTS assay showed the proliferation potential of SW620 cells after pc-DNA3.1-LY6D (pc-LY6D) transfection for 48 h (* *p* < 0.05, compared with pc-DNA3.1 transfection). (**F**) Flow cytometric analysis of Annexin V/PI staining to determine the percentage of apoptotic SW620SCs after pc-LY6D transfection for 48 h. (**G**) Representative photographs of SW620SCs that passed through the Matrigel after pc-LY6D transfection for 48 h in the Transwell invasion assay. (*** *p* < 0.001, compared with pc-DNA3.1 transfection). (**H**) Relative protein levels and normalization of c-Myc, Sox-2, Nanog, and Oct-4 detected via Western blotting after LY6D up-regulation (* *p* < 0.05, ** *p* < 0.01, compared with pc-DNA3.1 transfection).

As previously shown, LY6D expression was knocked down in HCT116SCs. We then determined the effects on HCT116SCs’ biological behavior. Therefore, the proliferation rate, percentage of apoptotic cells, and invasion abilities were examined after LY6D knockdown. MTS assay results showed that the proliferation rate of HCT116SCs decreased after LY6D knockdown when compared with that of the controls (Figure 3A). Flow cytometry results further showed that the proportion of apoptotic HCT116SCs increased after LY6D knockdown when compared with the control levels (Figure 3B). A transwell assay revealed that the invasion ability of HCT116SCs decreased after LY6D knockdown when compared with that of the controls (Figure 3C). Finally, Western blotting results showed that the down-regulation of LY6D decreased the expression of the stem cell markers c-Myc, Nanog, Sox-2, and Oct-4 significantly (Figure 3D).

### 2.4. Up-Regulation of LY6D Expression Increased the Proliferation, Invasion, and Stemness of SW620SCs

We also examined the proliferation rate, percentage of apoptotic cells, and invasion abilities after up-regulating LY6D expression. MTS assay results showed that the proliferation rate of SW620SCs increased after up-regulating LY6D expression when compared with that of the control (Figure 3E). However, the flow cytometry results showed that there was no difference of apoptosis between the pc-LY6D and pc-DNA3.1 groups (Figure 3F). The transwell assay revealed that the invasion ability of SW620SCs could increase after LY6D overexpression when compared with that in the control cells (Figure 3G). Moreover, Western blotting results showed the stem cell markers c-Myc, Nanog, Sox-2, and Oct-4 were significantly increased after LY6D up-regulation (Figure 3H).

### 2.5. LY6D Regulated CCSCs’ Proliferation, Apoptosis, and Invasion via the MAPK Pathway

**Figure 4:**
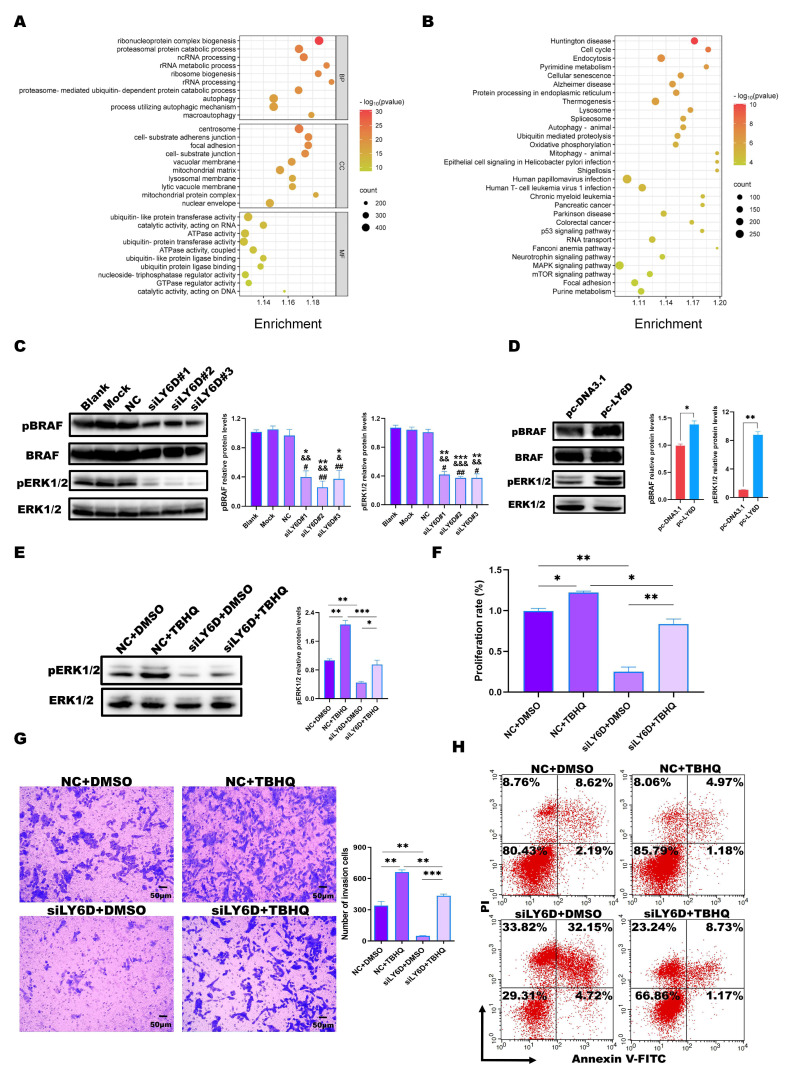
Bioinformatic analysis and validation of the regulatory effects of LY6D on colon cancer stem cells (CCSCs). (**A**) Gene Ontology (GO) enrichment analysis of differentially expressed genes (DEGs) following LY6D or control siRNA transfection for 48 h. (**B**) Kyoto Encyclopedia of Genes and Genomes (KEGG) enrichment analysis of DEGs following LY6D or control siRNA transfection for 48 h. (**C**) Western blotting results of pBRAF and pERK1/2 relative protein levels in HCT116SCs (stem cells) after siLY6D transfection for 48 h (* *p* < 0.05, ** *p* < 0.01, *** *p* < 0.001, compared with blank; ^&^ *p* < 0.05, ^&&^ *p* < 0.01, ^&&&^ *p* < 0.001, compared with mock; ^#^ *p* < 0.05, ^##^ *p* < 0.01, compared with negative control (NC)). (**D**) Western blotting results of pBRAF and pERK1/2 relative protein levels in SW620SCs after pc-LY6D transfection for 48 h (* *p* < 0.05, ** *p* < 0.01, compared with pc-DNA3.1 transfection). (**E**) Western blotting results of pERK1/2 relative protein levels in HCT116SCs after siLY6D transfection plus tert-butyl hydroquinone (TBHQ) treatment for 48 h assay (* *p* < 0.05, ** *p* < 0.01, *** *p* < 0.001). (**F**) MTS assay data for the proliferation potential of HCT116SCs after siLY6D transfection plus TBHQ treatment for 48 h assay (* *p* < 0.05, ** *p* < 0.01). (**G**) Representative photographs of HCT116SCs that passed through the Matrigel after siLY6D transfection plus TBHQ treatment for 48 h in the Transwell invasion assay (** *p* < 0.01, *** *p* < 0.001). (**H**) Flow cytometric analysis of Annexin V/PI staining to determine the percentage of apoptotic HCT116SCs after siLY6D transfection plus TBHQ treatment for 48 h.

We performed RNA sequencing using the siLY6D and NC groups to explore the mechanism by which the down-regulation of LY6D expression affects CCSCs. GO and KEGG enrichment analyses were conducted for the DEGs based on the *p*-values < 0.05 and a log_2_|FC| > 0.5 criteria. The GO enrichment analysis results showed that the cellular components associated with these DEGs were concentrated mainly in focal adhesions, the cell–substrate junction, and the cell–substrate adherens junction (Figure 4A). KEGG enrichment analysis results showed that the DEGs were mainly enriched in the MAPK pathway (Figure 4B). Furthermore, Western blotting results showed that the expression levels of pBRAF and pERK1/2, cascade kinases of the MAPK pathway, were reduced after LY6D knockdown (Figure 4C). Simultaneously, we verified the experimental conclusion from different perspectives. First, we validated the data by using SW620SCs. After up-regulating LY6D expression, we found that the expression levels of pBRAF and pERK1/2 increased significantly (Figure 4D). Furthermore, experiments comprising the knockdown and up-regulation of LY6D indicated that the MAPK pathway was downstream of LY6D.

Furthermore, ERK1/2-activator response experiments were performed, which revealed that treatment with TBHQ could partially restore the reduction in pERK1/2 levels caused by LY6D knockdown (Figure 4E). TBHQ was also able to partially restore the reduced proliferation rate and invasion abilities after LY6D knockdown (Figure 4F,G). Consistently, the effect of LY6D knockdown on the percentage of apoptotic HCT116SCs was suppressed upon TBHQ treatment (Figure 4H). The results indicate that LY6D can regulate CCSCs’ proliferation, apoptosis, and invasion via the MAPK pathway.

In addition, DUSP7, NEB, and RPS6KA1 were predicted to have simultaneous interactions with LY6D and ERK1/2 in the genemania database (http://genemania.org (accessed on 25 October 2023)) (Supplement Figure S1A), whereas the analysis in the KEGG database (https://www.kegg.jp/ (accessed on 25 October 2023)) showed that DUSP7, as a member of the MKP family, can cause the dephosphorylation of pERK 1/2 (Supplement Figure S1B). Therefore, LY6D might regulate ERK1/2 via DUSP7.

### 2.6. siLY6D-Mediated Inhibition of CC Xenograft Tumor Growth

**Figure 5:**
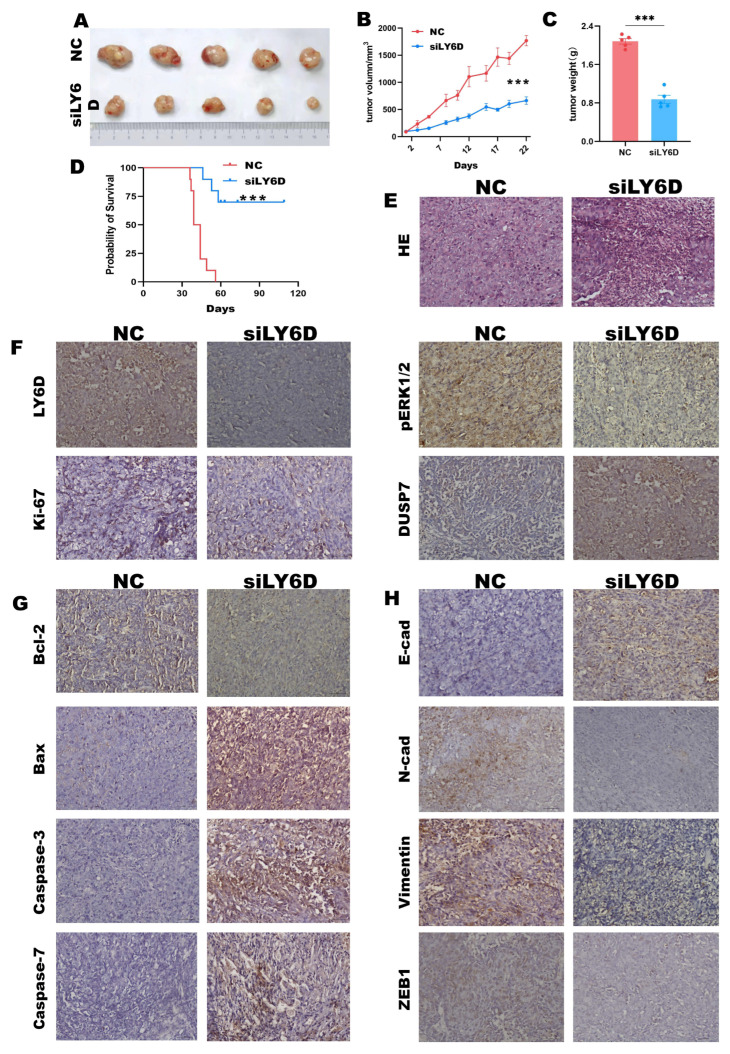
LY6D siRNA treatment suppresses xenograft tumor growth and prolongs mice survival rate. (**A**) Representative tumor photographs from HCT116SCs’ (stem cells) xenograft in mice following LY6D siRNA treatment for three weeks. (**B**) Tumor volumes of the HCT116SCs’ xenograft tumors from mice following LY6D siRNA treatment for three weeks (*** *p* < 0.001, compared with negative control (NC)). (**C**) Tumor weights of the HCT116SCs’ xenograft in mice following LY6D siRNA treatment for three weeks (*** *p* < 0.001, compared with NC). (**D**) Survival curve of mice with HCT116SCs’ xenograft after LY6D siRNA treatment for three weeks (*** *p* < 0.001, compared with NC). (**E**) Representative images of hematoxylin and eosin (HE) staining. (**F**) Representative immunohistochemistry (IHC) images of LY6D, pERK1/2, Ki67, and DUSP7. (**G**) Representative IHC images about apoptosis-related proteins. (**H**) Representative IHC images of EMT-related proteins.

We conducted nude mice experiments to determine how siLY6D affects CCSCs in vivo. The results revealed decreased solid tumor size and tumor weights in the group treated with siLY6D compared to those in the NC groups (Figure 5A–C). Furthermore, siRNA administration did not affect the body weights of the nude mice (Supplement Figure S2). Mice treated with siLY6D had a longer survival rate than the control (Figure 5D). HE staining of the resected solid tumor tissues showed that the tumors’ NC group had large nuclei and regular and tight cell arrangements; meanwhile, those in the siLY6D group exhibited a scrambled cell arrangement with inflammatory cell infiltration (Figure 5E). IHC staining showed that after siLY6D administration, the expression of LY6D (Figure 5F), pERK1/2 (Figure 5F), and the proliferation-related protein Ki67 (Figure 5F) were significantly down-regulated; the expression of DUSP7 (Figure 5F), which can dephosphorylate pERK1/2, was significantly down-regulated; the expression of Bcl-2, a protein that inhibits apoptosis, was significantly down-regulated; and levels of proteins that promote apoptosis, namely Bax, caspase-3, caspase-7, and caspase-9, were significantly increased (Figure 5G). The decreased expression of LY6D might inhibit the phosphorylation of pERK1/2 by increasing the expression of DUSP7, resulting in a decrease in proliferation and invasion and increase in apoptosis.

Moreover, under these conditions, the expression of the epithelioid protein E-cad was significantly up-regulated. In contrast, that of the interstitial-like proteins, N-cad, vimentin, and ZEB1, was significantly down-regulated (Figure 5H). In brief, the results demonstrated that LY6D knockdown can suppress colon tumor growth and improve the survival rate of experimental mice.

## 3. Discussion

The LY6 family genes were found to be located on chromosome 8q24.3, in which human cancer genes are frequently amplified, and researches have pointed out that some LY6 family members play key roles in various tumors [45,46]. LY6A is aberrant expressed in pituitary tumors and can be a tumor-initiating biomarker of lung cancer [47,48]; LY6E can serve as an independent prognostic factor for CC and is closely associated with the migration and invasion of CC [23,24]; and LY6K promotes the progression of bladder, ovarian, and breast cancers [25,26,27,28,29,30].

A member of the LY6 family, LY6D, contributes to the progression of basosquamous and prostate cancer [31,32]. It has already been reported that LY6D can be used as a prognostic biomarker of advanced prostate cancer associated with prostate CSCs [32]. Since CCSCs has a higher expression of LY6D, higher colony formation, and higher proliferation capability in our research, we investigated the effects of siLY6D on the proliferation, invasion, stemness, and apoptosis of CCSCs. The results showed that treatment of the CCSCs with siLY6D can decreases proliferation, invasion, and stemness and increase apoptosis. Studies have reported that the knockdown of the LY6 family member, LYNX1, reduces pMEK expression in the MAPK pathway [49], which links the LY6 family to the MAPK pathway. ERK1/2, also named MAPK1, is an important cell signaling molecule that mediates signal transduction into the nucleus and is an important regulator of cell proliferation, differentiation, apoptosis, etc. [50]. Relevant studies have shown that the activation of the ERK1/2 signaling pathway leads to the proliferation, survival, invasion, and drug resistance of tumor cells, as well as promotes the progression of CC tumors [51,52,53,54,55]. And, it has also been pointed out that BRAF mutations can activate the ERK1/2 signaling pathway, thereby promoting cell proliferation and survival [56]. Similar results were obtained using RNA sequencing in our research. Our results indicate that the DEGs were significantly enriched on the MAPK pathway in the LY6D knockdown groups when compared with those control groups. Our studies showed that the pBRAF and pERK1/2 of HCT116SCs were reduced significantly after LY6D knockdown; the pBRAF and pERK1/2 of SW620SCs were increased significantly after LY6D up-regulation. As the cascade kinases of the MAPK pathway, pBRAF and pERK1/2, are thought to promote the proliferation and migration of different cancer cells [57,58,59,60]. In addition, bioinformatics analysis predicted that DUSP7, as a protein co-expression with LY6D and ERK1/2, might be the target of LY6D. However, the detailed regulation mechanism needs further exploration.

Our in vivo experimental results also showed that xenograft IHC results from mice treated with siLY6D showed a lower expression of pERK1/2 and a higher expression of DUSP7 compared to the NC group. Overall, the results of the present study showed that LY6D can regulate CC via the MAPK pathway. Furthermore, in vivo results showed that the expression of the EMT marker, E-cad, was increased in siLY6D-treated mice, and N-cad, vimentin, and ZEB1 were decreased. This is similar to a previous finding where the knockdown of LY6E inhibited EMT in non-small-cell lung cancer cells [61]. To further confirm our experimental conclusions, we performed TBHQ response experiments. TBHQ, a putative ERK1/2 activator that has been reported in many studies, can promote the expression of pERK1/2 to increase tumor cell proliferation, migration, and invasion [62,63,64]. Our results showed that the knockdown of LY6D followed by TBHQ treatment can restore the diminishing proliferation and invasion and increase apoptosis of HCT116SCs due to the knockdown of LY6D.

Notably, LY6D knockdown not only significantly increased the apoptotic level of HCT116SC but also the necrosis level of HCT116SC. However, the levels of apoptosis and necrosis of SW620SC were not significantly changed when LY6D was overexpressed. Nevertheless, there are no current reports on the association of LY6 family members with tumor necrosis. Based on the above experimental results, the promotion of tumor necrosis by siLY6D may be regulated via other pathways, which should be investigated further via the subsequent experiments. Focusing on apoptosis and necrosis, we will explore the effects of ERK1/2 inhibitors on LY6D overexpression in SW620SCs. Furthermore, a thorough investigation about how LY6D affects cell apoptosis in CCSCs via the MAPK pathway will be performed. The results will provide a more comprehensive theoretical basis for diagnosing and treating CC, in addition to further theoretical support for the key role of LY6D in tumor development. Overall, the results of the in vivo and in vitro studies showed that LY6D could be used as a biomarker for CC and that siLY6D treatment might improve CC prognosis via the MAPK pathway.

## 4. Materials and Method

### 4.1. Cell Lines and Cell Culture

#### 4.1.1. CC Cell Lines

The CC cell lines Colo 205, HT-29, HCT116, SW480, and SW620 were purchased from the Cell Bank of the Chinese Academy of Sciences (CAS, Shanghai, China). They were cultured in an RPMI 1640 medium (Gibco, Thermo Fisher, Staley Rd., Grand Island, NY, USA) supplemented with 10% FBS (Gibco Thermo Fisher, Staley Rd., Grand Island, NY, USA). 

#### 4.1.2. CCSCs

CD44^+^CD326^+^HCT116 cells (HCT116SCs) and CD44^+^CD326^+^SW620 cells (SW620SCs) were cultured in a DMEM/F12 medium (Gibco, Thermo Fisher, Staley Rd., Grand Island, NY, USA) supplemented with 20 ng/mL epidermal growth factor (Invitrogen, Thermo Fisher, Staley Rd., Grand Island, NY, USA), 20 ng/mL basic fibroblast growth factor (Invitrogen, Thermo Fisher, USA), and 1 × B27 optimized serum-free supplement (Gibco, Thermo Fisher, Staley Rd., Grand Island, NY, USA).

### 4.2. Cell Sorting

According to the manufacturer’s instructions, immunomagnetic bead separation was conducted using a CELLection™ Biotin Binder Kit (Invitrogen, Thermo Fisher, VNO, Lithuania). First, 4.0 × 10^7^ CC cells were collected and then incubated with the pre-prepared anti-CD44 antibody (Invitrogen, Thermo Fisher, USA)-coated Dynabeads^®^ at 4 °C for about 20 min. Subsequently, the unbound cells were removed and the bound cells were released from the anti-CD44 antibody complex by mixing the complex with a reconstituted Release Buffer (DNase I), incubating for 15 min at room temperature with gentle tilting and rotation, pipetting thoroughly with a 200 μL pipette 5–10 times to maximize cell release, placing the tube with the released cells in a magnet for 2 min, and then transferring the supernatant with the released cells into a new tube, where finally, free CD44^+^ CC cells were generated and obtained. The collected CD44^+^ CC cells were incubated with the pre-prepared anti-CD326 antibody (Invitrogen, Thermo Fisher, USA)-coated Dynabeads^®^ at 4 °C for about 20 min. Then, the unbound cells were removed, the bound cells were released from the anti-CD326 antibody complex, and the same method as the CD44^+^ CC cells were obtained, where finally, free CD44^+^ CD326^+^ CC cells were generated and obtained.

### 4.3. Cell Transfection

LY6D siRNA#1 (siLY6D#1, 5′-GCAACTGCAAGCATTCTGTGGTCTG-3′), LY6D siRNA#2 (siLY6D#2, 5′-GCCAGCTCTCGCTTCTGCAAGACCA-3′), LY6D siRNA#3 (siLY6D#3, 5′-TCTGGTGAAGAAGGACTGTGCGGAG-3′), and negative control siRNA (NC, STEALTH RNAI NEG CTL HI GC) were provided by Thermo Fisher Scientific Co., Ltd. pc-DNA3.1 and pc-DNA3.1-LY6D (pc-LY6D) were provided by PPL (Public Protein/Plasmid Library, Nanjing, China).

The seeding of cells (5.0 × 10^5^ cells/well) was performed in six-well plates, and transfection was performed using the Hieff TransTM Liposomal Transfection Reagent (Yeasen, Shanghai, China). The cells were collected after 48 h and used in subsequent experiments.

### 4.4. Cell Proliferation

The MTS kit (Promega Corporation, Madison, WI, USA) was used as the testing reagent for the proliferation assay. The cells were transfected and seeded in 96-well plates at 1.0 × 10^4^ cells/well. The proliferation rate was detected after 48 h using the MST solution (20 μL/well). The optical density was measured at 450 nm.

### 4.5. Colony Formation Assay

For the colony formation assay, each group was seeded 5.0 × 10^3^ cells in a 10 cm plate, cultured in 10 mL RPMI 1640 medium (Gibco, Thermo Fisher, Staley Rd., Grand Island, NY, USA) supplemented with 10% FBS (Gibco Thermo Fisher, Staley Rd., Grand Island, NY, USA), and every 3 days the medium was refreshed. After 14 days, 4% paraformaldehyde (Biosharp, Hefei, China) was used for cell fixation, 1% crystal violet (Solarbio Life Sciences, Beijing, China) was used to stain the colonies.

### 4.6. Tert-Butyl Hydroquinone (TBHQ) Response Assay

TBHQ (60 µM, MedChemExpress, Monmouth Junction, NJ, USA), an ERK1/2 phosphorylation activator, was added to the cells after transfection for 6 h. The TBHQ-treated cells were collected 42 h later and used in subsequent experiments.

### 4.7. Cell Apoptosis

Cell apoptosis was tested 48 h post-transfection using the FITC Annexin V Apoptosis Detection Kit I (Beton Dickinson and Company, San Diego, CA, USA). For each group, the cells (1.0 × 10^5^) were collected, washed with cold 1× PBS twice, and then stained in the dark with Annexin V-FITC (5 μL) and PI (5 μL) solutions, at 20~25 °C, each for about 15 min, measuring the samples within one hour.

### 4.8. Cell Invasion

Invasion assays were performed using Transwell plates (8 μm pore size, Corning, One Riverfront Plaza, NY, USA) plus a thin layer Matrigel (Corning, One Riverfront Plaza, NY, USA). The cells (1.0 × 10^5^) in 100 µL DMEM/F12 without FBS were seeded in the upper chamber. Chemo-attractant DMEM/F12 (500 μL) supplemented with 10% FBS was placed into the lower chamber. A solution of 4% paraformaldehyde (Biosharp, Hefei, China) was used for cell fixation and crystal violet (Solarbio Life Sciences, Beijing, China) was used to stain the cells on membranes after 48 h. Then, the upper chamber cells were erased. Finally, invading cells from three fields were selected randomly, photographed, and counted.

### 4.9. RNA-Seq and Bioinformatics Analysis

#### 4.9.1. RNA-Seq

The total RNA of the NC and siLY6D groups was extracted using TRIzol (Invitrogen, Thermo Fisher, USA). Sequencing libraries were generated using the NEBNext^®^ UltraTM RNA Library Prep Kit for Illumina^®^ (NEB, Ipswich, MA, USA). To attribute sequences, for each sample, index codes were added. The RNA-seq was performed on a cBot Cluster Generation System using the TruSeq PE Cluster Kit v3-cBot-HS (Illumina, San Diego, CA, USA).

#### 4.9.2. GO and KEGG Enrichment Analysis

Gene Ontology (GO) enrichment analysis of Differentially Expressed Genes (DEGs) between NC and siLY6D was performed using the clusterProfiler R package. GO terms, for BB, CC, and MF, with *p* < 0.05 and a fold change (log_2_|FC|) > 0.5, were considered to be statistically significantly enriched.

Kyoto Encyclopedia of Genes and Genomes (KEGG) is a database of biological systems (http://www.genome.jp/kegg/ (accessed on 21 September 2022)). KEGG enrichment analysis of DEGs between NC and siLY6D was performed using the clusterProfiler R package. KEGG pathways with *p* < 0.05 and a fold change (log_2_|FC|) > 0.5 were considered to be statistically significantly enriched.

### 4.10. Western Blotting

Total proteins of each group were extracted using a RIPA lysis buffer (DINGGUO, Beijing, China) supplemented with protease inhibitors (Beyotime Biotechnology, Shanghai, China). Proteins were separated using 10% sodium dodecyl sulfate polyacrylamide gel (SDS-PAGE) and transferred onto a polyvinylidene fluoride (PVDF) membrane. The membrane was blocked with 5% skim milk at 20~25 °C for 1 h and incubated with the following antibodies overnight at 4 °C to detect target proteins: anti-LY6D antibody (1:1000, Thermo Fisher); anti-BRAF antibody (1:1000, CST); anti-pBRAF antibody (1:1000, CST); anti-ERK1/2 antibody (1:1000, CST); anti-pERK1/2 antibody (1:1000, CST); and anti-GAPDH antibody (1:1000, Beyotime Biotechnology). Subsequently, the membrane was washed three times with a 1 × TBS buffer containing 0.1% Tween-20 (TBST) and then incubated with an anti-rabbit IgG secondary antibody (1:1000, Beyotime Biotechnology, China) for 1 h at 20~25 °C, followed by rewashing three times in 1 × TBST. Finally, the target proteins were detected using a super ECL detection reagent (Yeasen, Shanghai, China), and the membranes were viewed under a Tanon Imaging System (Tanon, Shanghai, China).

### 4.11. Animals

Six-week-old, specific pathogen-free male, athymic BALB/c nude mice were provided by Beijing HFK Bioscience Co., Ltd (Beijing, China). Animal ethics and experimental procedures were performed according to protocols approved by the Animal Care Committee of Jilin University (No. 20210065).

### 4.12. Xenograft Assay

For the in vivo tumor xenograft assay, HCT116SCs (5.0 × 10^5^) were injected into the right flank of each mouse subcutaneously. The mice were divided into two groups treated with 50 μL 0.9% NaCl containing either 5 nmol NC siRNA or LY6D siRNA at random, intratumorally, three times a week for three weeks. Tumor sizes were measured manually using a vernier caliper every other day where we defined that a represents the tumor length and b represents the tumor width, where the tumor volume is then calculated as (a × b^2^)/2 [65]. Electronic balance was used to measure the mice weights every other day and the tumors weights at the time that the mice were sacrificed.

### 4.13. Hematoxylin-Eosin (HE) and Immunohistochemistry (IHC) Staining

Tumor samples were fixed with 4% paraformaldehyde and embedded in paraffin. For the hematoxylin-eosin (HE) staining, tumor tissue sections were dewaxed with xylene, rehydrated with an aqueous solution with decreasing ethanol concentrations, and stained with HE.

For immunohistochemistry (IHC) staining, deparaffinized sections were incubated in 3% H_2_O_2_ for about 30 min to quench the endogenous peroxidase. Slides were microwaved in 10 mM citrate buffer (pH 6.0) for approximately 15 min to retrieve the antigen. Subsequently, the slides were incubated overnight at 4 °C with the target protein antibodies: anti-LY6D antibody (1:100, Thermo Fisher, Staley Rd., Grand Island, NY, USA); anti-DUSP7 (1:100, absin); anti-pERK1/2 antibody (1:100, CST); anti-Ki67 antibody (1:100, CST); anti-Bcl2 antibody (1:100, CST); anti-Bax antibody (1:100, CST); anti-Caspase3 antibody (1:100, Abcam, Discovery Drive, Cambridge, UK); anti-caspase7 antibody (1:100, Abcam, Discovery Drive, Cambridge, UK); anti-E-cad antibody (1:100, CST); anti-N-cad antibody (1:100, CST); anti-vimentin antibody (1:100, CST); and anti-ZEB1 antibody (1:100, CST). Following incubation with the biotin-conjugated secondary antibody and streptavidin solution, a DAB Horseradish Peroxidase Color Development Kit (Beyotime Biotechnology, Shanghai, China) was used to develop the sections’ color.

### 4.14. Statistical Analysis

GraphPad 9.5.1 (GraphPad Software, San Diego, CA, USA) was used for statistical analysis. The data are expressed as mean ± SEM. Student’s *t*-test and ANOVA analysis were used to analyze the variances between groups. *p* < 0.05 was considered to be statistically significant.

## 5. Conclusions

**Figure 6:**
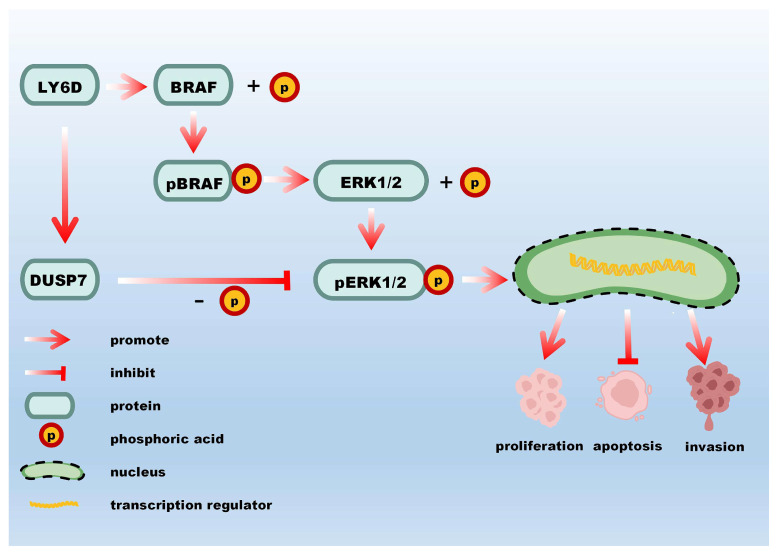
A schematic representation of the molecular mechanism by which LY6D affects CCSCs’ proliferation, apoptosis, and invasion.

In this study, LY6D was found to contribute to the progression of CC and regulate the proliferation, invasion, and apoptosis of CCSCs via the MAPK pathway (Figure 6). Our research demonstrates that LY6D could serve as a potential target and therapeutic marker for CC treatment.

## Data Availability

All datasets used and/or analyzed during this study are available from the corresponding author upon reasonable request.

## Supplementary Materials

The following supporting information can be downloaded at: https://www.mdpi.com/article/10.3390/molecules28237776/s1, Figure S1: Bioinformatics analysis of the interaction between LY6D and ERK1/2. (A) Genemania database analysis revealed NEB, RPS6KA1, DUSP7 interact with both LY6D and ERK1/2 (also called MAPK1). (B) KEGG database analysis showed DUSP7 inhibits ERK1/2 phosphorylation. Figure S2: Body weights change of the CC xenograft after siLY6D administration.

## Abbreviations

CC, colon cancer; CCSCs, colon cancer stem cells; CSC, cancer stem cell; DEG, differentially expressed gene; DUSP7, dual specificity phosphatase 7; EMT, epithelial–mesenchymal transition; ERK, extracellular signal-regulated kinase; FBS, fetal bovine serum; HE, hematoxylin and eosin; GPI, glycosyl-phosphatidylinositol; IHC, immuno-histochemistry; LY6D, lymphocyte antigen 6 family member D; PI, propidium Iodide; PVDF, Polyvinylidene fluoride; NC, negative control siRNA; PBS, phosphate-buffered saline; siLY6D, LY6D siRNA; TBHQ, tert-butylhydroquinone; TBS, Tris-buffer solution.
