# Supplementary figures and images for: Silencing LY6D Expression Inhibits Colon Cancer in Xenograft Mice and Regulates Colon Cancer Stem Cells’ Proliferation, Stemness, Invasion, and Apoptosis via the MAPK Pathway
Source: Molecules. 2023 Nov 25;28(23):7776. doi: 10.3390/molecules28237776 (PMC10708431; doi:10.3390/molecules28237776)

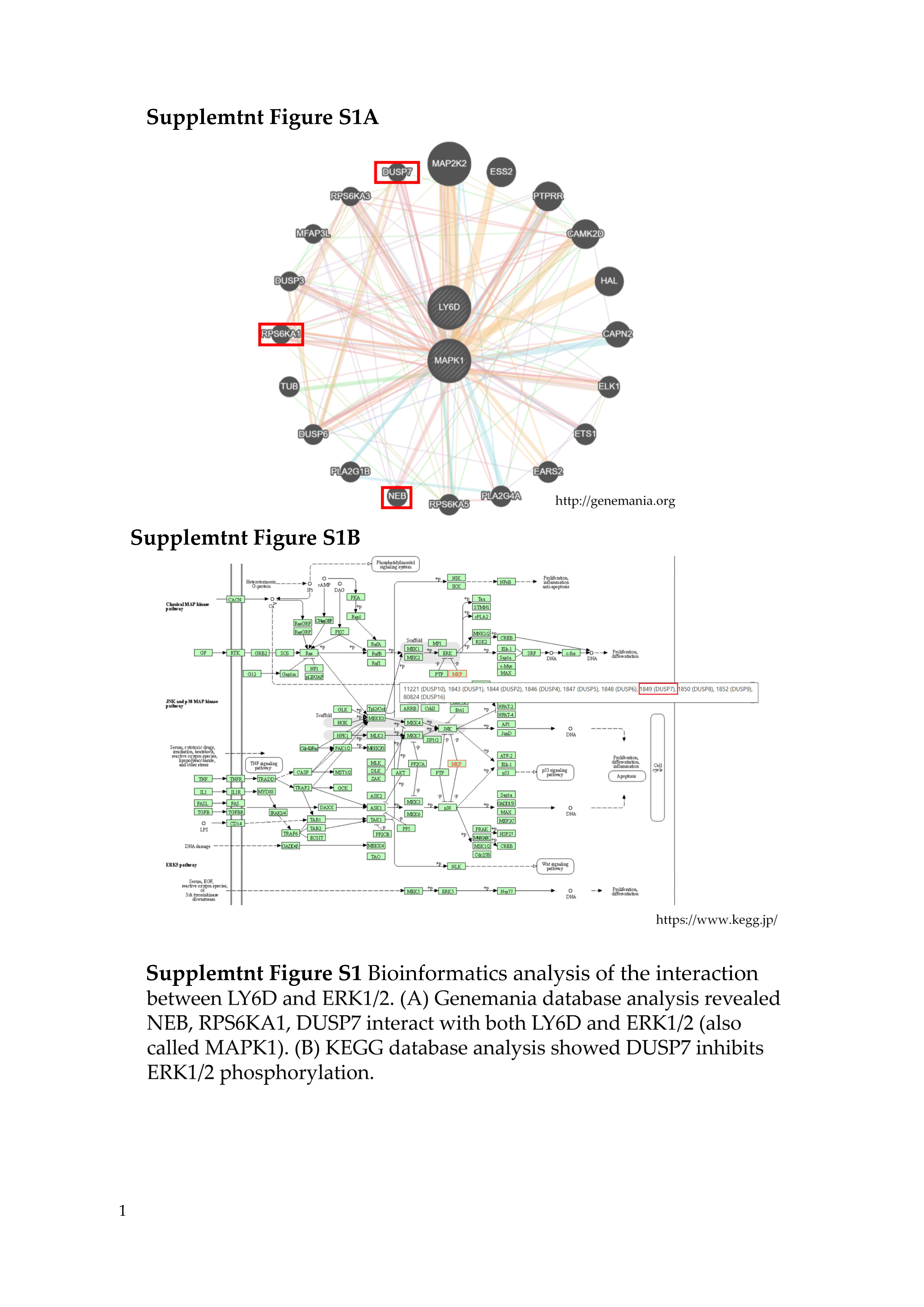

Supplement: Supplementary file 1 [file molecules-28-07776-s001.zip › Supplement Figure S1.tif]

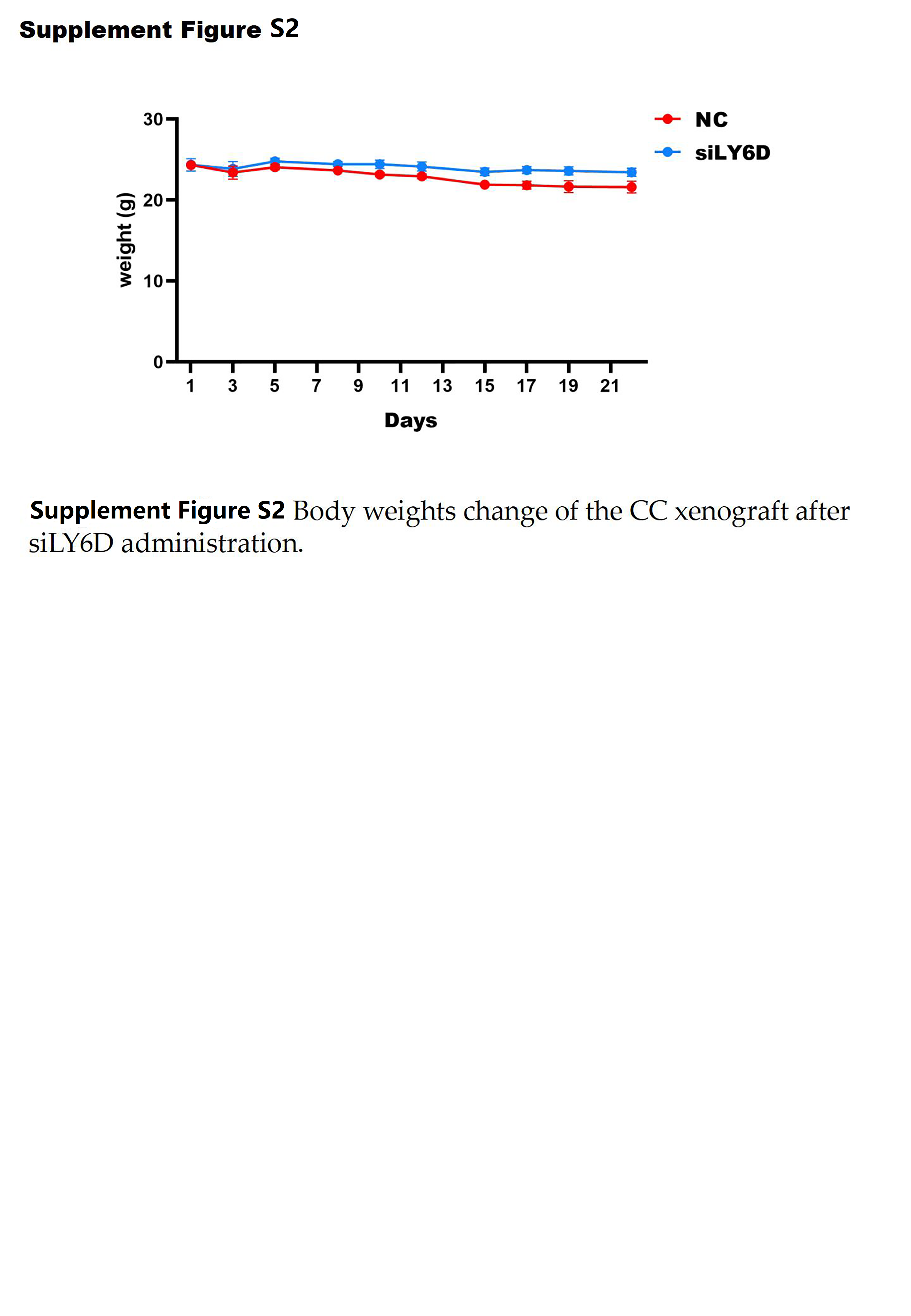

Supplement: Supplementary file 1 [file molecules-28-07776-s001.zip › Supplement Figure S2.tif]
